# Inhibition of Gap Junction Formation Prior to Implantation of Bone Marrow-Derived Mesenchymal Cells Improves Function in the Ischemic Myocardium

**DOI:** 10.3390/ijms24119653

**Published:** 2023-06-02

**Authors:** Santipongse Chatchavalvanich, Robert A. Boomsma, Jack M. Tietema, David L. Geenen

**Affiliations:** 1 Department of Basic Biomedical Sciences, Dr. William M. Scholl College of Podiatric Medicine, Rosalind Franklin University of Medicine and Science, North Chicago, IL 60064, USA; s.chatchavalvanich@rosalindfranklin.edu; 2 Biology Department, Trinity Christian College, Palos Heights, IL 60463, USA; bob.boomsma@trnty.edu; 3 Michigan State University College of Human Medicine, Grand Rapids, MI 49503, USA; tietemaj@msu.edu; 4 Physician Assistant Studies Department, College of Health Professions, Grand Valley State University, Grand Rapids, MI 49503, USA

**Keywords:** stem cell, ischemic myocardium, gap junction, connexin

## Abstract

Bone marrow-derived mesenchymal stem cells (BM-MSC) are reported to induce beneficial effects in the heart following ischemia, but a loss of these cells within hours of implantation could significantly diminish their long-term effect. We hypothesized that early coupling between BM-MSC and ischemic cardiomyocytes through gap junctions (GJ) may play an important role in stem cell survival and retention in the acute phase of myocardial ischemia. To determine the effect of GJ inhibition on murine BM-MSC in vivo, we induced ischemia in mice using 90 min left anterior descending coronary artery (LAD) occlusion followed by BM-MSC implantation and reperfusion. The inhibition of GJ coupling prior to BM-MSC implantation led to early improvement in cardiac function compared to mice in which GJ coupling was not inhibited. Our results with in vitro studies also demonstrated increased survival in BM-MSCs subjected to hypoxia after inhibition of GJ. While functional GJ are critical for the long-term integration of stem cells within the myocardium, early GJ communication may represent a novel paradigm whereby ischemic cardiomyocytes induce a “bystander effect” when coupled to newly transplanted BM-MSC and thus impair cell retention and survival.

## 1. Introduction

Myocardial ischemia and infarction (MI) result in the loss of cardiomyocytes and are one of the major causes of morbidity and mortality worldwide [1]. The restoration of blood flow is necessary to salvage the ischemic cardiomyocytes; however, the reperfusion and reoxygenation of the tissue are usually associated with the exacerbation of the injury and significant inflammatory response, termed “reperfusion injury” [2].

The concept that the heart is a terminally differentiated organ has recently been challenged. Several studies have shown that the heart contains a population of self-renewing progenitor cells that can differentiate into and replenish cardiomyocytes in both physiologic and pathologic conditions [3,4,5,6]. Several sources of cells have been identified and studied for use in cell-based cardiac therapy and previous studies have shown that bone marrow-derived stem cell administration or mobilization following myocardial infarction can improve cardiac function [7,8,9]. Moreover, a recent systematic review of clinical trials examining the effectiveness of adult bone marrow-derived stem cells in treatment of acute MI showed that stem cell treatment significantly improves global heart function with a positive correlation with stem cell dose [10]. Our laboratory and several others have also demonstrated improved function following administration of stem cells both in animal models and in transplanted human hearts [11,12,13,14,15]. Several studies have demonstrated that mesenchymal stem cells (MSCs) selectively home to the injured organ regardless of tissue type [16]. This has also been shown in work from our laboratory where murine bone marrow-derived MSCs (mBM-MSCs) injected intravascularly are recruited to the ischemic heart [11].

A significant loss of progenitor cells from the myocardium has been observed within hours after transplantation and likely diminishes the long-term benefits of cardiac regenerative therapy [17,18,19,20]. The rapid loss of stem cells after transplantation could be a bystander effect of exposure to dying cardiomyocytes. Recent studies demonstrate increasingly diverse roles for gap junctions and describe them as intercellular signaling complexes that alter cell functions and produce bystander effects in coupled cells [21,22,23,24]. The role of gap junction channels in the propagation of cell death has been indicated in both physiological and pathological conditions. For example, Cusato et al. demonstrated that gap junctions transmit apoptotic signals in developing retina [25]; Maass et al. implicated the involvement of gap junction communication in infarct expansion and showed that preventing gap junctions from closing resulted in increased infarct size in their model [26]; and Qiao et al. found that stem cells given intramyocardially rapidly decreased in number within the first 24 h of their administration, with a corresponding increase in percentage of apoptotic cells [27]. The mechanism by which this occurs has not been elucidated, but we speculated that it involves, at least in part, the coupling of BM-MSCs to cells in the target organ and transmission of cellular metabolites, molecules and ions between target and engrafting cells.

Electrical integration is an essential component in smooth propagation of the action potential, and in regenerative medicine, a great deal of attention has been paid to promoting gap junction formation as a means to integrate newly administered stem cells into the existing myocardium. In contrast, very little is known about the detrimental effects of early cell–cell coupling on stem cell retention within the ischemic myocardium despite evidence that gap junctions mediate a bystander effect. The overall hypothesis of this study is that gap junction channels may be at least partially responsible for the propagation of cell death from ischemic cardiomyocytes transferred to stem cells and participate in their loss after transplantation.

## 2. Results

### 2.1. Hypoxic Myocytes Induced Apoptosis and Cell Death in Co-Cultured BM-MSCs Which Could Be Attenuated by Carbenoxolone (CBX)

**Figure 1:**
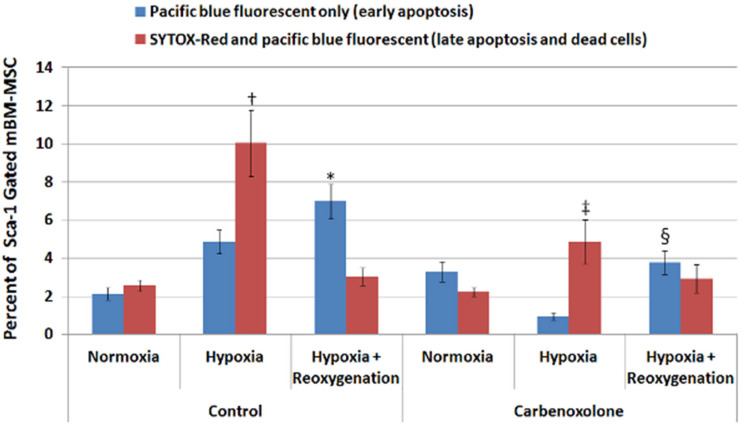
Hypoxia and reoxygenation which increased cell death could be attenuated by the use of carbenoxolone. Aggregate data from the cell culture experiments. Hypoxia and hypoxia +reoxygenation increased the number of mBM-MSCs stained positive for dead (SYTOX-Red) and apoptotic (Annexin V) markers, respectively. Carbenoxolone, a gap junction inhibitor, significantly attenuated the increase in number of dead and apoptotic mBM-MSCs in the corresponding group. (Data are presented as mean ± S.E.M.; *n* = 4–6; † and *: statistically significant when compared to the respective normoxia control group, *p* < 0.05; ‡ and §: statistically significant when compared to the respective hypoxia and hypoxia + reoxygenation control group, *p* < 0.05).

An HL-1 murine cardiac muscle atrial cell monolayer was cultured in normoxic or hypoxic conditions for four hours. At the end of four hours, BM-MSCs were added to the monolayers and cultured together either in the same conditions (Normoxia and Hypoxia, respectively) or switched from hypoxic to normoxic conditions (Hypoxia +Reoxygenation) for an additional two hours. Another set of experiments was conducted in the presence of 100 µM of CBX, a non-selective gap junction blocker. After the coculture, the cells were lifted; stained with anti-Sca-1 antibody, SYTOX-Red dead cell stain and Pacific Blue-conjugated anti-Annexin V; and analyzed by flow cytometry. Sca-1 was used as a marker to gate for BM-MSC due to the high percentage (~80%) of expression in MSC compared to HL-1 (~1%). There was a very low baseline number of cells stained positive for SYTOX-Red or Annexin V in BM-MSC from normoxic control cocultures (Figure 1). Coculturing with HL-1 in hypoxic conditions significantly increased the number of dead and late apoptotic BM-MSCs indicated by positive SYTOX-Red staining, while Annexin V-positive BM-MSCs showed a slight increase, albeit not statistically significant. Interestingly, BM-MSCs cocultured with hypoxic HL-1 in normoxic condition (hypoxia + reoxygenation) showed a significant increase in the number of early apoptotic cells stained positive for Annexin V. The presence of the non-specific gap junction blocker carbenoxolone (CBX) in the media of the coculture significantly attenuated the increase in SYTOX-Red-positive (dead and late apoptotic cells) and Annexin V-positive BM-MSCs in hypoxia and hypoxia–reoxygenation group, respectively.

### 2.2. CBX Treatment Did Not Affect BM-MSC Proliferation

**Figure 2:**
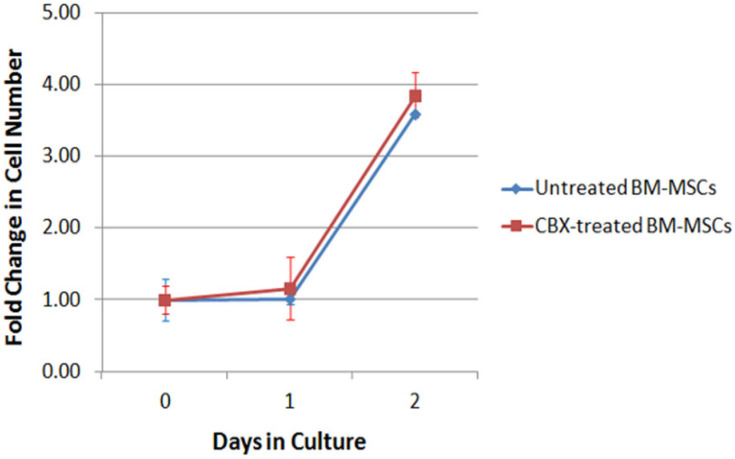
CBX treatment does not affect BM-MSC proliferation. Cells in each group were counted and all data normalized to the number of cells at the start of the culture (1 × 10^5^) and presented as mean ± S.E.M. (*n* = 3 per group). No significant difference was observed between groups at any time point (*p* > 0.05).

We conducted in vitro studies to determine whether CBX affects the proliferative capacity of the transplanted BM-MSCs in culture. BM-MSCs were treated overnight with 100 µM of CBX or vehicle, then plated on 6-well plates at 1 × 10^5^ cells/well, which is the same number we used for our subsequent in vivo study. Cell number was measured using an automatic cell counter. After one day in culture, the cells in both groups did not increase from the day of plating (1.02 vs. 1.16 fold original cell number for control and CBX-treated BM-MSCs groups, respectively (Figure 2)). On day two, BM-MSCs in both groups showed a four-fold increase in cell number from the day of plating but no statistically significant difference between two groups.

### 2.3. CBX-Treated BM-MSCs Improved Cardiac Function after Ischemia-Reperfusion Injury

**Figure 3:**
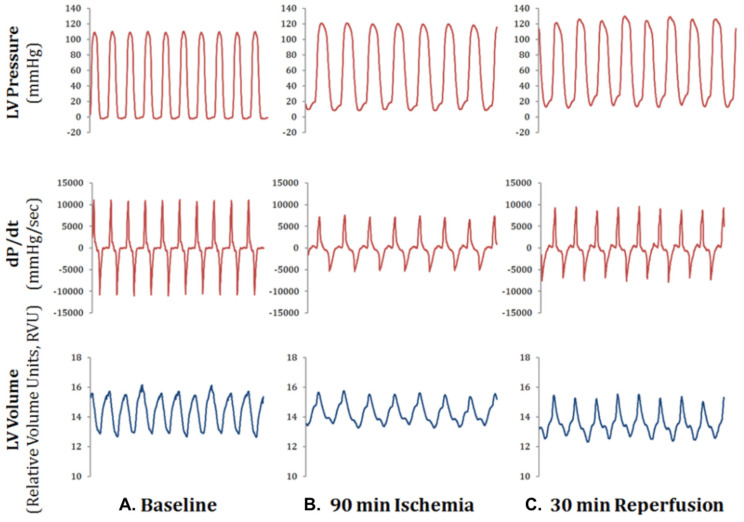
Changes in cardiac function following ischemia-reperfusion. Sample hemodynamic data from one animal undergoing ischemia-reperfusion showing LV pressure, dP/dt, and LV volume at baseline (panel **A**), after 90 min of ischemia (panel **B**), and after 30 min of reperfusion (panel **C**). Each panel represents data recorded in 1 s.

Heart rate, LV pressure and LV volume were recorded at baseline, ischemia (coronary artery ligation) and reperfusion in a subset of (*n* = 3) mice to determine the changes in cardiac function prior to our mBM-MSC injection studies. We chose 90 min of ischemiadue to the observed depressed cardiac function at this time point (Figure 3). Partial recovery of cardiac function was observed after 30 min of reperfusion (Figure 3).

**Figure 4:**
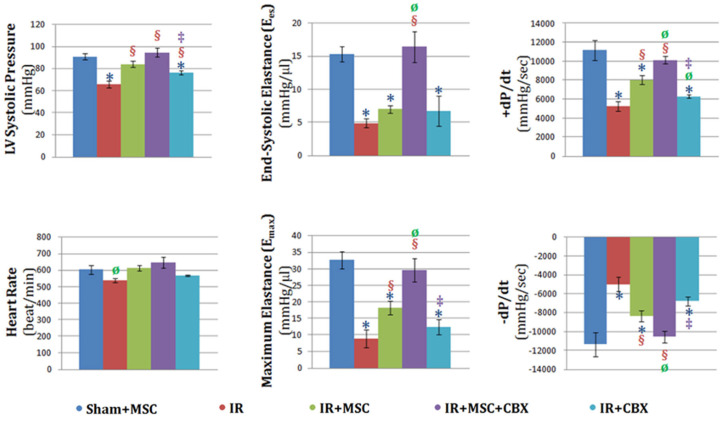
Injection of CBX-treated BM-MSCs in the myocardium attenuated the loss of cardiac function observed following ischemia-reperfusion. Sham+MSC: sham-operated group receiving MSCs; IR: group with ischemia-reperfusion without MSC; IR+MSC: group with ischemia-reperfusion receiving MSCs; IR+MSC+CBX: group with ischemia-reperfusion receiving CBX-treated MSCs; IR+CBX: group with ischemia-reperfusion receiving vehicle with CBX. Data are presented as means ± S.E.M.; *n* = 4, 3, 10, 5 and 4, animals per group, respectively; *: statistically significant when compared to respective Sham+MSC group, *p* < 0.05; §: statistically significant when compared to respective IR group, *p* < 0.05; Ø: statistically significant when compared to respective IR+MSC group, *p* < 0.05; ‡: statistically significant when compared to respective IR+MSC+CBX group, *p* < 0.05.

All mice undergoing ischemia-reperfusion received injections of either BM-MSCs, CBX-treated BM-MSCs, CBX alone or vehicle and were and were allowed to recover for 24 h after which time left ventricular catheterization and functional analysis occurred (Figure 4).

Aggregate hemodynamic data were analyzed in PVAN software (ver 3.5) to calculate LVelastance, end-systolic pressure–volume relationship (ESPVR), end-diastolic pressure–volume relationship (EDPVR), heart rate, LV pressure and dP/dt (Figure 4). Heart rate did not vary among experimental groups except in the ischemia/reperfusion (IR) group, in which it was significantly lower than sham animals receiving BM-MSCs (Sham+MSC). LV pressure was significantly decreased in IR animals compared to Sham+MSC. The decrease in pressure observed in the IR group was attenuated by treatment with BM-MSCs (IR+MSC). This attenuation was further enhanced significantly in animals that received CBX-treated BM-MSCs (IR+MSC+CBX). The vehicle containing CBX (IR+CBX) failed to emulate the protective effect observed in IR+MSC+CBX. End-systolic elastance (E_es_) and maximum elastance (E_max_) were significantly decreased in IR animals compared to Sham+MSC. As expected, BM-MSC treatment improved E_max_ significantly above the level observed in IR group, albeit not completely back to the Sham+MSC level. The decreased E_es_ and E_max_ were rescued to levels comparable to Sham+MSC, when the animals were given CBX-treated BM-MSCs. Similar to our earlier observation, CBX alone did not affect E_es_ and E_max_ in the animals. Consistent with elastances, maximum and minimum pressure derivatives (±dP/dt) decreased significantly in the IR group. Treatment with BM-MSCs attenuated the reduction in these measures of contractile function. The pretreatment of BM-MSCs with CBX further improved the pressure derivatives and restored them to levels comparable to those of control animals. On the other hand, CBX alone did not display the same beneficial effects.

### 2.4. CBX-Treated mBM-MSC Retention in the Heart 24 h after Ischemia-Reperfusion Injury

**Figure 5:**
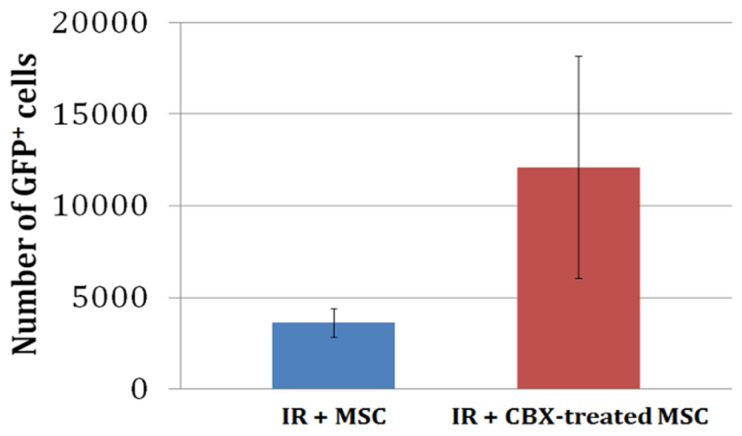
Retention of transplanted MSCs in the heart. Number of green fluorescent protein^+^ (GFP) cells from whole heart digests in the untreated (IR+MSC) vs. treated cells (IR+CBX-treated MSC) detected by flow cytometry analysis 24 h following IR injury. *n* = 5 (untreated) and 11(CBX-treated), respectively. *p* > 0.05.

In a separate set of experiments, untreated or CBX-treated GFP^+^ BM-MSCs were injected into the myocardium following IR. The animals were allowed to recover for 24 h after which their hearts were excised and enzymatically digested. The whole heart digests were then analyzed for the number of GFP-positive BM-MSCs. The average number of GFP^+^ cells in whole heart digests from animals receiving CBX-treated BM-MSCs (12,926 ± 6926) tended to be greater than that in the untreated group (3620 ± 786). However, this difference was not statistically significant due to the larger variability in the CBX-treated BM-MSCs group (Figure 5).

### 2.5. CBX-Treated mBM-MSC Did Not Alter Area-at-Risk or Infarct Size at 24 h

**Figure 6:**
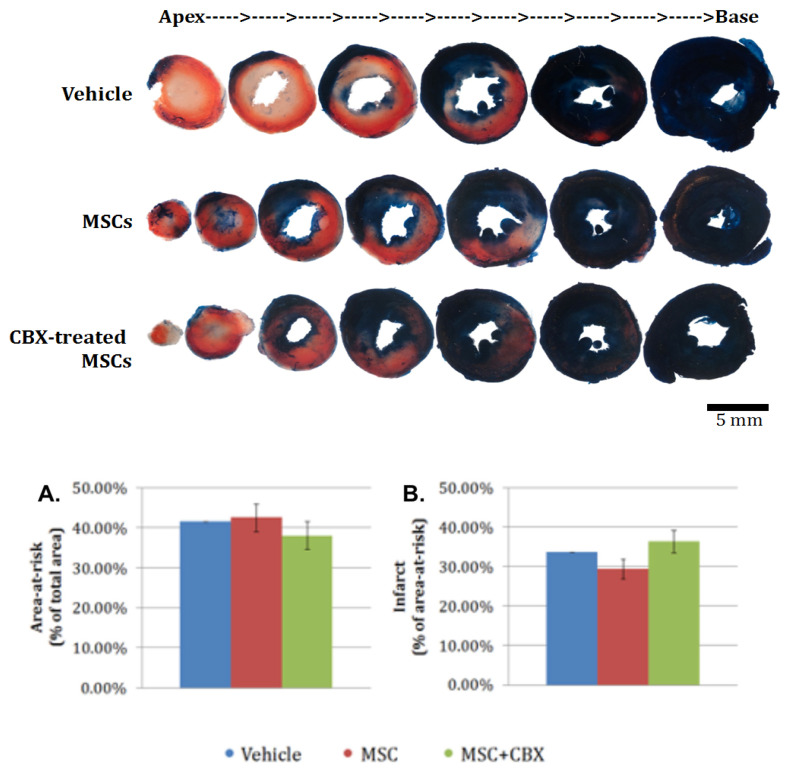
Treatment with CBX-treated MSCs does not affect area-at-risk or infarct size at 24 h following ischemia-reperfusion injury. Top: Representative image of heart slices from vehicle, MSC, and CBX-treated MSC groups 24 h after ischemia-reperfusion. Bottom: Mean + S.E.M. are represented for the area-at-risk (panel **A**) and infarct size (panel **B**) from the experiments. *n* = 1, 4, and 6 for vehicle, MSC, and CBX-treated MSC, respectively. *p* > 0.05. ANOVA was used to compare the MSC vs. MSC+CBX groups.

To determine whether BM-MSCs treatment would result in any detectable cardiac remodeling at this early time point, the area-at-risk and infarct size were assessed in the hearts of the control, BM-MSCs, and CBX-treated BM-MSCs groups by triphenyltetrazolium chloride (TTC) staining. Images of heart slices were analyzed as detailed in the method section. We observed no statistically significant difference in either area-at-risk (panel A) or infarct size (panel B) among the groups (Figure 6).

## 3. Discussion

Our study is the first, to our knowledge, to examine the role of gap junction intercellular communication in propagating the loss of BM-MSC exposed to hypoxic myocytes both in culture and in the whole heart. The roles of hypoxia and reoxygenation in causing cell injury have been extensively studied and well established in different cells, including cardiomyocytes. The number of transplanted progenitor cells has been shown to decrease dramatically within hours of administration and the percentage of transplanted cells peaked at 24 h after transplantation [27]. Cells that become ischemic could trigger detrimental responses in adjacent cells which have not been affected by the original insult, a phenomenon termed “bystander effect” which has been extensively described and studied in radiation injury [28]. Gap junctions have been suggested to play an important role in propagating the signals involved in ionizing radiation-associated bystander effect. Therefore, we proposed that communication through gap junctions could be one of the mechanisms contributing to the loss of transplanted stem cells in ischemic myocardium and impairing their therapeutic efficacy. Consistent with this hypothesis, data from our laboratory were used to examine the interaction of mBM-MSCs with HL-1 cardiac cells [29,30]. We demonstrated that MSCs rapidly establish intercellular coupling with cardiac myocytes [29]. Furthermore, BM-MSCs demonstrated an uptake of gap junction permeant calcein from HL-1 cells within 4 h of co-culture. This effect was partially attenuated when the BM-MSCs were treated with oleamide, a gap junction inhibitor [30].

In the present study, co-culturing with hypoxic HL-1 cardiomyocytes elicited a significant increase in the number of dead and apoptotic BM-MSCs. The observed difference between the ratio of dead to apoptotic cells could be due to the fact that in the hypoxia group, the HL-1s had been under hypoxia for a total of 6 h, while hypoxia lasted for only 4 h in the hypoxia/reoxygenation group, resulting in more damage to the HL-1. In the hypoxia group, there were more SYTOX-Red-positive dead BM-MSCs than Annexin V-positive early apoptotic BM-MSCs, while in Hypoxia/Reoxygenation group, the majority of dying BM-MSCs was only stained positive for Annexin V, suggesting that they underwent early apoptosis. The presence of CBX in the co-cultures of BM-MSCs and hypoxic HL-1 cardiocytes dramatically reduced the numbers of dead and apoptotic BM-MSCs. CBX non-selectively blocks gap junction channels. This result, taken together with our earlier work demonstrating gap junction communication within hours following co-culture [29], suggests the important role of gap junction communication in triggering BM-MSCs death and apoptosis when exposed to hypoxic cardiocytes.

Gap junction channels play a seminal role in organ development, cell growth, cell differentiation and the maintenance of cell phenotypes within specific organs and system niches [21,31,32,33,34,35]. Specifically, several studies also report a role for gap junction proteins, connexins, in regulating bone marrow cell proliferation and differentiation [36,37,38]. Recent studies demonstrate increasingly diverse roles for gap junctions and describe them as intercellular signaling complexes that alter cell functions and propagate bystander cell damage in coupled cells [21,22,23,24,25]. The postulated mechanism suggests a passage of small molecules that act as a “bystander signal” between cells through gap junction channels. Due to the constraint in gap junction channel size, the estimated limit is 2 nm in diameter and in the range of 1–10 kDa [28,31,32,33,34,35,36,37,38,39]. Although this study did not identify the mediator responsible for the death of BM-MSCs, we hypothesize that one of the plausible candidates is Ca^2+^. During ischemia, energy depletion affects Na^+^/K^+^ and Ca^2+^ pumps and consequently causes increases in cytosolic Ca^2+^, which in turn induces a spectrum of deleterious sequelae [40]. Ca^2+^ accumulates in mitochondria and triggers opening of mitochondrial permeability transition pores that further promote mitochondrial depolarization and results in the release of cytochrome c from the inner membrane of mitochondria to the cytosol. Cytochrome c release sequentially results in the oligomerization of Apaf-1, formation of an apoptosome, activation of caspase-9, and activation of effector caspases [41].

We examined whether the blocking of gap junctions in BM-MSCs prior to administration could enhance their protective effects on cardiac function in the heart after ischemia-reperfusion injury. Our results clearly indicated an improvement in the protective effects bestowed by CBX-treated BM-MSCs as compared to both untreated and control BM-MSC-treated hearts as demonstrated by the improvement in cardiac hemodynamic parameters compared to the sham-operated hearts. This beneficial effect conferred by CBX-treated BM-MSCs even surpassed that observed in the hemodynamic function of control BM-MSC-treated animals, indicating an increased efficacy associated with CBX pre-treatment.

We further investigated whether this improvement in hemodynamics was associated with an increased cell retention following ischemia/reperfusion in vivo. We hypothesized that there would be greater cell retention in the CBX-treated BM-MSC hearts because of an increased number of viable BM-MSCs. This was supported by our in vitro data which demonstrated a significant decrease in early apoptosis of CBX-treated BM-MSCs compared to untreated BM-MSCs under hypoxia/reoxygenation conditions (Figure 1). Although we observed a strong trend toward increased retention of CBX-treated BM-MSCs compared to untreated BM-MSCs (12,926 ± 6926 vs. 3620 ± 786, respectively) under ischemia/reperfusion conditions in vivo, the data also exhibited large variability and did not reach statistical significance. There are a number of reasons why these results differed between our in vitro and in vivo data. One possible explanation is that the number of BM-MSCs injected into the myocardium were sufficient to produce beneficial functional effects but insufficient to demonstrate significant retention. Our in vitro studies used a 5:1 ratio of HL-1 cardiocytes to BM-MSCs in co-culture, while our injections in vivo were conducted with 1 × 10^5^ BM-MSCs in the whole heart. Under these conditions, the ratio of endogenous cardiocytes to BM-MSCs would be closer to a 10:1 ratio. It is also unclear whether CBX-treated cells, which were prevented from coupling with endogenous cardiocytes, may have responded differently to the ischemic environment than the untreated cells that presumably formed gap junctions with ischemic endogenous cardiocytes. Furthermore, the elapsed time following injection and analysis of surviving BM-MSCs (24 h) may not have been of a sufficient length to detect significant differences in retention between the CBX-treated and untreated BM-MSCs. It is noteworthy, however, that the number of CBX-treated BM-MSCs retained in the ischemic/reperfused hearts was sufficient to enhance cardiac function and is consistent with earlier studies reported by Clifford that the number of MSCs used in clinical trials is directly proportional to their effect on cardiac function [10].

Similar to our results reported above for the retention of injected stem cells, there were no significant differences in area-at-risk or infarct size between the vehicle alone, BM-MSC untreated or CBX-treated BM-MSC groups 24 h after injection. Ventricular remodeling following ischemia is an evolving process that occurs over 4–6 weeks in mice. Changes that might be undetectable at this early time with CBX-treated BM-MSCs could lead to decreased necrosis and scar tissue by the time the infarct had healed. This is substantiated by earlier published work from our laboratory that BM-MSCs synthesize and secrete multiple paracrine factors that are able to affect MSC migration, promote angiogenesis and reduce apoptosis [30]. Both monocyte chemoattractant protein-1 (MCP-1) and PI3-kinase are involved in this protective effect, although they are independent of each other. It is likely that multiple pro-survival factors in addition to MCP-1 are secreted by BM-MSCs which act on divergent intracellular signaling pathways. It is plausible then that viable CBX-treated BM-MSCs did express pro-survival factors including MCP-1 and PI3-kinase, as previously shown, but that the length of time between the injection of these cells in vivo and the measurement of area at risk (24 h) did not allow for sufficient time to elapse to observe reductions in infarct size or area-at-risk.

Because GJs are integral to cardiac muscle function, the long-term inhibition of GJs in transplanted BM-MSCs may disrupt the therapeutic effects in a clinical setting or trigger potentially dangerous arrhythmias [33]. Additionally, previous research has found that increased GJ formation between BM-MSCs and cardiomyocytes improves the long-term effects of BM-MSC therapy [28]. It is possible that gap junction formation between cardiomyocytes and transplanted BM-MSCs may be deleterious to the survival of these cells in the acutely ischemic environment, but ultimately, coupling between the two cell types is necessary to ensure integration and electrical coupling.

## 4. Materials and Methods

### 4.1. Animals

Mice were housed in the AAALAC-accredited Biological Resources Laboratory at University of Illinois at Chicago and maintained in accordance with the National Institutes of Health Guide for the Care and Use of Laboratory Animals. Experimental protocols were approved by the Institutional Animal Care and Use Committee at University of Illinois at Chicago.

### 4.2. Bone Marrow Mesenchymal Stem Cell Harvest

BM-MSCs were isolated from C57BL/6 mice (Jackson Laboratory, Bar Harbor, ME) as previously described [11,30]. Briefly, tibia and femur were stripped of muscle and placed in ice-cold phosphate-buffered saline (PBS) + 2% fetal bovine serum (FBS). The epiphyseal ends were removed, and the bones were centrifuged at 4000× *g* for 1 min in a modified microfuge tube. The bone marrow cells were suspended in ice-cold PBS + 2% FBS, passed through a 70 μm filter and counted with a hemocytometer.

Filtered bone marrow cells were suspended in PBS + 2% FBS + 0.1 g/L phenol red and enriched for lineage negative (Lin^−^) cells using the SpinSep system (StemCell Technologies). The cells were incubated with Murine Progenitor Enrichment Cocktail (anti-CD45, anti-CD45R, anti-CD11b, anti-Gr-1, anti-TER119 and anti-7/4; Stem Cell Technologies, Vancouver, CA) on ice for 30 min, washed and incubated with dense particles on ice for 20 min. The cells were layered on density medium, centrifuged at 1200× *g* for 10 min and the layer of cells at the density medium/PBS interface was collected, washed and counted.

Enriched bone marrow cells were placed on tissue culture-treated plates at a density of 0.1 × 10^6^ cells/cm^2^ in murine MesenCult media (StemCell Technologies) with 100 U/mL of penicillin, 100 μg/mL of streptomycin, and 0.25 μg/mL of amphotericin B added. The media was changed after 48 h and adherent cells were maintained in culture with twice weekly media changes. After 4 wk, the confluent cells were detached with trypsin and split 3:1. Lin^−^ MSCs were characterized for surface antigens using flow cytometry.

### 4.3. Adenoviral-Mediated Fluorescent Labeling of Mesenchymal Stem Cells

BM-MSCs were plated 1 × 10^5^ cells in a 100 mm tissue culture dish in MesenCult media with 100 U/mL of penicillin, 100 μg/mL of streptomycin, and 0.25 μg/mL of amphotericin B. Custom-made recombinant adenovirus expressing green fluorescent protein (GFP) under the control of a CMV promoter (AdGFP) was added to the culture at the time of plating at multiplicity of infection (MOI) of 15. After 48 h, the cells were lifted with trypsin and subjected to fluorescent-activated cell sorting to enrich for cells that highly express GFP for subsequent experiments.

### 4.4. Flow Cytometry Analysis and Fluorescent-Activated Cell Sorting

Cells were harvested by trypsinization with 0.25% trypsin for 5 min at 37 °C and incubated in flow cytometry buffer (PBS with 2 mM EDTA and 0.25% BSA) with 1 μL of anti-mouse IgG (Sigma, St. Louis, MO, USA); 1:50) for 5 min on ice. Cells were then incubated with fluorescent-conjugated antibodies for 1 h on ice. The antibodies used included anti-Sca-1-APC (eBioscience, San Diego, CA, USA); 1:50), SYTOX-Red dead cell stain (Invitrogen), and anti-Annexin V-Pacific Blue (Invitrogen, Waltham, MA, USA). Cells were washed, resuspended in flow cytometry buffer and analyzed (Cyan ADP, Beckman Coulter or LSRFortessa, BD Biosciences, East Rutherford, NJ, USA) in the Flow Cytometry Laboratory of University of Illinois at Chicago Research Resources Center. The data were analyzed by either Summit (Beckman Coulter, Brea, CA, USA) or FACSDiva (BD Biosciences, East Rutherford, NJ, USA) software (ver 9.0). For cell sorting, cells were prepared as described above and then sorted by MoFlo High Speed Sorter (Dako-Cytomation, Santa Clara, CA, USA). The sorted cells were plated or frozen for later use.

### 4.5. Co-Culture Experiments

HL-1 murine atrial myocytes were seeded at 1 × 10^5^ in a 6-well plate and placed in either DMEM with glucose under normoxic conditions or in DMEM with 2-deoxy-glucose at pH 6.2 in a sealed chamber (1% O_2_, 5% CO_2_, balance N_2_) for 4 h. Then, 2 × 10^4^ Lin^−^ murine BM-MSCs were seeded on the HL-1 monolayer, and the co-cultures were returned to the cell incubation chamber. Two co-culture groups continued under their original conditions, i.e., normoxic conditions (normoxia) and conditions (hypoxia). In a third group, the original hypoxic media was replaced with control DMEM, and the incubation continued under normoxic conditions (hypoxia/reoxygenation). A non-selective gap junction inhibitor carbenoxolone (CBX) was added to half of the wells from each of the groups at a final concentration of 100 μ immediately prior to the co-culture [33]. Cells remained in co-culture for an additional 2 h, after which they were lifted and labeled with fluorescent probes (anti-Sca-1, SYTOX-Red and anti-Annexin V, as described above) for flow cytometry analysis. Samples were gated by their Sca-1-APC signal to select only BM-MSC for cell death analysis.

### 4.6. Coronary Ligation and Cell Injection

Wild-type mice (C57BL/6; Jackson Laboratories, Bar Harbor, ME) were sedated with etomidate (10 mg/kg body weight, i.p.) and maintained with 1.5% isoflurane delivered through a vaporizer with 100% O_2_ connected serially to a rodent ventilator with the tidal volume set at 0.2 to 0.3 mL/min (based on body weight) and a respiratory rate of 135 per min. A left thoracotomy was performed, the heart was exposed, and the left anterior descending coronary artery was ligated with an 8-0 prolene suture, as previously described [11]. A suture was passed under the coronary artery without ligation in sham-operated mice. The mice were maintained on isoflurane for 90 min following the coronary occlusion after which the suture was removed, and the ischemic area was reperfused. Mice were assigned into the following groups: sham-operated control with 1 × 10^5^ BM-MSCs injection (Sham+MSC), coronary occlusion and reperfusion with vehicle injection (IR), coronary occlusion and reperfusion with 1 × 10^5^ BM-MSCs injection (IR+MSC), coronary occlusion and reperfusion with 1 × 10^5^ CBX-treated BM-MSCs injection (IR+MSC+CBX) and coronary occlusion and reperfusion with vehicle containing CBX (IR+CBX). In mice with coronary occlusion, the apical myocardium represented the ischemic area and was injected with 10 μL of injection buffer (Ca^2+^/Mg^2+^-free PBS + 2 mM EDTA + 0.25% BSA) with or without 1 × 10^5^ BM-MSCs at the time of reperfusion. All BM-MSCs used were labeled with GFP using adenoviral transduction and sorted to enrich for cells that were highly expressing GFP. BM-MSCs in CBX-treated groups were incubated with 100 μM of CBX overnight prior to the injection. All of the data and sample collections were made 24 h after ischemia-reperfusion.

### 4.7. Hemodynamic Measurements

Hemodynamic measurements were taken as previously described using an ultra-miniature pressure–volume catheter (1.4 French; SPR-839; Millar Instruments) to obtain heart rate, LV systolic and diastolic pressure, the pressure derivatives (±dP/dt) and pressure–volume loop [11,42]. The hemodynamic data obtained were analyzed using PVAN software package (Millar Instruments, Houston, TX) between different groups: Sham+MSC (*n* = 4), IR (*n* = 3), IR+MSC (*n* = 10), IR+MSC+CBX (*n* = 5) and IR+CBX (*n* = 4).

### 4.8. Whole Heart Digests

To estimate the relative number of BM-MSCs retained in the hearts at 24 h following cell injection, enzymatic digestion of the whole heart was performed using a method modified from O’Connell et al. and analysis by flow cytometry [43]. The hearts were cannulated and perfused retrograde at 37 °C with perfusion buffer (113 mM of NaCl, 4.7 mM of KCl, 0.6 mM of KH_2_PO_4_, 0.6 mM of Na_2_H_2_PO_4_, 1.2 mM of MgSO_4_, 0.032 mM of phenol red, 12 mM of NaHCO_3_, 10 mM of KHCO_3_, 10 mM of HEPES, 30 mM of taurine, 10 mM of 2,3-butanedioone monoxime, 5.5 mM of glucose at pH 7.4) for 4 min followed by digestion buffer (perfusion buffer with 0.1 mg/mL of liberase TM (Roche Applied Science, Penzberg, Germany) and 0.14 mg/mL of trypsin + 12.5 µN CaCl_2_) for 7–10 min. The hearts were removed from the cannula and minced in the digestion buffer at 37 °C until completely digested. Digestion was terminated by adding an equal volume of stop buffer (perfusion buffer + 10% bovine calf serum + 12.5 µM of CaCl_2_). Cells were centrifuged at 1000× *g* for 5 min and the buffer was replaced with flow cytometry buffer for subsequent flow cytometry analysis (see above). Flow cytometry measurements were gated for the BM-MSC population by size, and granularity and the number of GFP-positive cells were recorded.

### 4.9. Infarct Size Measurement by Tetrazolium Staining

Infarct size and area-at-risk were determined by a method modified from Redel et al. [44]. Twenty-four hours after ischemia-reperfusion, the hearts were harvested from the mice. For each heart, the aorta was cannulated and infused with 3 mL of ice-cold PBS. The coronary artery was then ligated with an 8-0 prolene suture at the exact location of the previous suture. Evans blue dye (1 mL; 0.1 g/mL) was injected retrograde into the aorta and perfused the heart excluding the left coronary artery bed. The heart was then removed from the cannula, placed in a chilled acrylic heart matrix with 1.0 mm transverse section slice intervals (Astor Industries, Lakemba, Australia) and cooled at −20 °C for 20 min. Subsequently, the heart was cut into 6–7 1 mm thick transverse slices. The heart slices were sandwiched between glass cover slips and incubated at 37 °C for 25 min in 2% 2,3,5-triphenyltetrazolium chloride (TTC; Sigma, St. Louis, MO, USA) dissolved in 0.1 *M* Na_2_HPO_4_/NaH_2_PO_4_ buffer adjusted to pH 7.4 and then fixed in 10% buffered formalin at 4 °C overnight. The heart slices were placed between two glass slides for imaging.

The images of slices from each heart were taken at 3.2× magnification with a Stemi 2000C stereomicroscope (Carl Zeiss, Baden Wurttemberg, Germany) using a D300s digital camera (Nikon, Tokyo, Japan) and Clearshot 600 Digital Camera Adapter System (Alexis Scientific). The images were then analyzed using the Mobile Infarct Tool program to measure the area stained by Evans blue (blue), TTC (red) and neither of the dyes (white) [45]. Data from all the slices from each heart were combined to provide total area of area-at-risk and infarct. Area-at-risks are reported as percent of area at risk to total myocardial area, and infarct sizes are reported as percent of infarcted area to area-at-risk area.

### 4.10. Murine Bone Marrow Mesenchymal Stem Cell Proliferation Assay

Cell proliferation assays were performed on BM-MSCs and CBX-treated BM-MSCs. Cells were plated in 6-well plates at 1 × 10^5^ cells/well. The number of cells at days 0, 1 and 2 were counted using Cellometer Auto 2000 Cell Counter (Nexcelom Bioscience, Waltham, MA, USA).

### 4.11. Statistical Analysis

Data from the experiments were organized and analyzed in Microsoft Excel (Redmond, WA, USA). Values are presented as means ± SEM and *n* is the number of experiments. Statistical differences among means were analyzed by one-way ANOVA and post-hoc *t*-tests with the criteria for statistical significance set at *p* ≤ 0.05.

## 5. Conclusions

In conclusion, a great deal of attention has been paid to promoting gap junction formation as a means to achieve functional integration of newly administered stem cells into the existing myocardium [18,46]. Our results suggest that while gap junction formation is necessary to achieve electrical integration of transplanted progenitor cells into the host tissue, early coupling through these same gap junctions could have pernicious effects on transplanted BM-MSCs and that disrupting this early communication could prove beneficial to stem cell survival following myocardial ischemia. Further evaluation of the feasibility of modulating the temporal sequence of gap junction formation between cells and isolating the role of specific isoforms of connexin that contribute to the formation of gap junctions will help to elucidate the role that these channels play in stem cell therapy for the heart.

## Data Availability

The data used to support the findings of this study are available from the corresponding author and can be shared on Dropbox.
